# European pollen reanalysis, 1980–2022, for alder, birch, and olive

**DOI:** 10.1038/s41597-024-03686-2

**Published:** 2024-10-03

**Authors:** Mikhail Sofiev, Julia Palamarchuk, Rostislav Kouznetsov, Tamuna Abramidze, Beverley Adams-Groom, Célia M. Antunes, Arturo H. Ariño, Maximilian Bastl, Jordina Belmonte, Uwe E. Berger, Maira Bonini, Nicolas Bruffaerts, Jeroen Buters, Paloma Cariñanos, Sevcan Celenk, Valentina Ceriotti, Athanasios Charalampopoulos, Yolanda Clewlow, Bernard Clot, Aslog Dahl, Athanasios Damialis, Concepción De Linares, Letty A. De Weger, Lukas Dirr, Agneta Ekebom, Yalda Fatahi, María Fernández González, Delia Fernández González, Santiago Fernández-Rodríguez, Carmen Galán, Björn Gedda, Regula Gehrig, Carmi Geller Bernstein, Nestor Gonzalez Roldan, Lukasz Grewling, Lenka Hajkova, Risto Hänninen, François Hentges, Juha Jantunen, Evgeny Kadantsev, Idalia Kasprzyk, Mathilde Kloster, Katarzyna Kluska, Mieke Koenders, Janka Lafférsová, Poliana Mihaela Leru, Agnieszka Lipiec, Maria Louna-Korteniemi, Donát Magyar, Barbara Majkowska-Wojciechowska, Mika Mäkelä, Mirjana Mitrovic, Dorota Myszkowska, Gilles Oliver, Pia Östensson, Rosa Pérez-Badia, Krystyna Piotrowska-Weryszko, Marje Prank, Ewa Maria Przedpelska-Wasowicz, Sanna Pätsi, F. Javier Rodríguyez Rajo, Hallvard Ramfjord, Joanna Rapiejko, Victoria Rodinkova, Jesús Rojo, Luis Ruiz-Valenzuela, Ondrej Rybnicek, Annika Saarto, Ingrida Sauliene, Andreja Kofol Seliger, Elena Severova, Valentina Shalaboda, Branko Sikoparija, Pilvi Siljamo, Joana Soares, Olga Sozinova, Anders Stangel, Barbara Stjepanović, Erik Teinemaa, Svyatoslav Tyuryakov, M. Mar Trigo, Andreas Uppstu, Mart Vill, Julius Vira, Nicolas Visez, Tiina Vitikainen, Despoina Vokou, Elżbieta Weryszko-Chmielewska, Ari Karppinen

**Affiliations:** 1 Finnish Meteorological Institute, Helsinki, Finland; 2 Center of Allergy & Immunology, Tbilisi, Georgia; 3 University of Worcester, School of Science and Environment, Worcester, UK; 4 University of Évora, School of Health and Human Development, Department of Medical and Health Sciences & Institute of Earth Sciences - ICT, Évora, Portugal; 5 University of Navarra, Biodiversity and Environment Institute, Pamplona, Spain; 6 Department of Otorhinolaryngology, Medical University of Vienna, Vienna, Austria; 7 Departament de Biologia Animal, Biologia Vegetal i Ecologia, Universitat Autònoma de Barcelona (UAB), Bellaterra, Spain; 8 Institut de Ciència i Tecnologia Ambientals (ICTA-UAB), Universitat Autònoma de Barcelona, Bellaterra, Spain; 9 University of Innsbruck, Department of Botany, Innsbruck, Austria; 10 Department of Hygiene and Health Prevention, Agency for Health Protection of Metropolitan Area of Milan (ATS), Milan, Italy; 11 Mycology and Aerobiology, Sciensano, Brussels, Belgium; 12 Center of Allergy & Environment (ZAUM), Member of the German Center for Lung Research (DZL), Technical University and Helmholtz Center Munich, Munich, Germany; 13 Department of Botany, University of Granada, Granada, Spain; 14 Andalusian Institute for Earth System Research (IISTA-CEAMA), University of Granada, Granada, Spain; 15 Bursa Uludag University, Faculty of Arts and Science, Department of Biology, Aerobiology Laboratory, 16059 Görükle-Bursa, Türkiye; 16 Department of Ecology, School of Biology, Faculty of Sciences, Aristotle University of Thessaloniki, Thessaloniki, Greece; 17 Health, air quality, & UK pollen forecasting, UK Met Office, Exeter, UK; 18 Federal Office of Meteorology and Climatology MeteoSwiss, Zurich, Switzerland; 19 Department of Biological and Environmental Sciences, University of Gothenburg, Gothenburg, Sweden; 20 Department of Pulmonology, Leiden University Medical Center, Leiden, the Netherlands; 21 Palynological Laboratory, Swedish Museum of Natural History, Stockholm, Sweden; 22 Sciences Faculty, University of Vigo, Ourense, 32002 Spain; 23 Biodiversity and Environmental Management, University of León, León, Spain; 24 Institute of Atmospheric Sciences and Climate-CNR, Bologna, Italy; 25 Department of Construction, School of Technology, University of Extremadura, Avda. de la Universidad s/n, Cáceres, Spain; 26 Inter-University Institute for Earth System Research (IISTA), International Campus of Excellence on Agri-food (ceiA3), University of Cordoba, Cordoba, Spain; 27 Zabludovicz Center for Autoimmune Diseases, Sheba Medical Center, Ramat Gan, Israel; 28 Pollen Laboratory, Department of Biological and Environmental Sciences, University of Gothenburg, Gothenburg, Sweden; 29 Laboratory of Aerobiology, Department of Systematic and Environmental Botany, Faculty of Biology, Adam Mickiewicz University, Poznan, Poland; 30 Czech Hydrometeorological Institute, Prague, Czech Republic; 31 Unit of Immunology-Allergology, Centre Hospitalier de, Luxembourg, Luxembourg; 32 South Karelia Allergy and Environment Institute, Imatra, Finland; 33 College of Natural Sciences University of Rzeszow, Rzeszow, Poland; 34 The Asthma and Allergy Association, Roskilde, Denmark; 35 Institute of Biology, College of Natural Sciences University of Rzeszow, Rzeszow, Poland; 36 Elkerliek Helmond, Helmond, Netherlands; 37 Regional Public Health Office department of medical microbiology, bratislava, Slovakia; 38 Clinical Department 5, Carol Davila University of Medicine, Bucharest, Romania; 39 Allergology Research Laboratory, Colentina Clinical Hospital, București, Romania; 40 Department of the Prevention of Environmental Hazard, Allergology and Immunology, Medical University of Warsaw, Warsaw, Poland; 41 Biodiversity Unit, University of Turku, Turku, Finland; 42 National Center for Public Health and Pharmacy, Budapest, Hungary; 43 “Aeroallergen Monitoring Centre ““AMoC”, Department of Immunology and Allergy, Allergy, Poland; 44 Medical University of Lodz, Lodz, Poland; 45 HUS Helsingin yliopistollinen sairaala, Jyväskylä, Finland; 46 Serbian Environmental Protection Agency, Belgrade, Serbia; 47 Jagiellonian University Medical College, Department of Clinical and Environmental Allergology, Kraków, Poland; 48 French Aerobiological Monitoring Network (RNSA), Brussieu, France; 49 University of Castilla-La Mancha, Institute of Environmental Sciences, Toledo, Spain; 50 Department of Botany and Plant Physiology, Subdepartment of Aerobiology, University of Life Sciences in Lublin, Lublin, Poland; 51 Icelandic Institute of Natural History, Akureyri, Iceland; 52 Department of Biology, NTNU, Trondheim, Norway; 53 Allergen Research Center, Warsaw, Poland; 54 Department of Pharmacy, National Pirogov Memorial Medical University, Vinnytsia, Ukraine; 55 Department of Pharmacology, Pharmacognosy and Botany, Faculty of Pharmacy, Complutense University of Madrid, Madrid, Spain; 56 Department of Biology Animal, Plant Biology and Ecology, University of Jaén, Jaén, Spain; 57 University Institute of research in Olive Groves and Olive Oils, University of Jaén, Jaén, Spain; 58 University Hospital Brno, Brno, Czech Republic; 59 Masaryk University, Brno, Czech Republic; 60 Vilnius University Siauliai Academy, Siauliai, Lithuania; 61 National Laboratory of Health, Environment and Food, Maribor, Slovenia; 62 Faculty of Biology, Moscow State University, Moscow, Russia; 63 Faculty of Biology, Shenzhen MSU -BIT University, Shenzhen, China; 64 Retired from Faculty of Pharmacy of the Belarusian State Medical University, Minsk, Belarus; 65 BioSense Institute Research Institute for Information Technologies in Biosystems, University of Novi Sad, Novi Sad, Serbia; 66 Stiftelsen NILU - Stiftelsen Norwegian Institute for Air Research, Kjeller, Norway; 67 University of Latvia, Riga, Latvia; 68 Laboratory of Aerobiology at Teaching Institute of Public Health dr. Andrija Štampar, Zagreb, Croatia; 69 Estonian Environmental research Institute (under Estonian Environmental Research Centre), Tartu, Estonia; 70 Department of Botany and Plant Physiology, University of Malaga, Malaga, Spain; 71 Université de Lille, CNRS, UMR, 8516, LASIRE - Laboratoire de Spectroscopie pour les Interactions, la Réactivité et l’Environnement, F-59000 Lille, France

**Keywords:** Environmental impact, Plant ecology, Scientific data

## Abstract

The dataset presents a 43 year-long reanalysis of pollen seasons for three major allergenic genera of trees in Europe: alder (*Alnus*), birch (*Betula*), and olive (*Olea*). Driven by the meteorological reanalysis ERA5, the atmospheric composition model SILAM predicted the flowering period and calculated the Europe-wide dispersion pattern of pollen for the years 1980–2022. The model applied an extended 4-dimensional variational data assimilation of *in-situ* observations of aerobiological networks in 34 European countries to reproduce the inter-annual variability and trends of pollen production and distribution. The control variable of the assimilation procedure was the total pollen release during each flowering season, implemented as an annual correction factor to the mean pollen production. The dataset was designed as an input to studies on climate-induced and anthropogenically driven changes in the European vegetation, biodiversity monitoring, bioaerosol modelling and assessment, as well as, in combination with intra-seasonal observations, for health-related applications.

## Background & Summary

Airborne pollen released by anemophilous plants during their flowering season can cause significant allergic manifestations impairing public health, especially in combination with other air pollutants and/or weather phenomena^1–5^. The health impact of pollen has exhibited an upward trend, with only a very low fraction of the population being concerned in the 1960s. In the late-1990s – early-2000s the prevalence of allergic rhinitis (AR) in Europe was estimated at ~15%^6^; with a range of 11–32% deduced by a study of 10 European countries^7^. In the 2010-s, the European Community Respiratory Health Survey established the prevalence of AR to be from 4% to 32%^8^. Recently, a thorough review indicated the range of 1% - 40% and reported a gradual increase of prevalence in already heavily affected countries^9^. The rise of AR cases coincided with an onset of asthma epidemics. The topic got particular attention due to a recent outbreak of thunderstorm-related asthma attacks^10^.

The increase of the prevalence of pollinosis and other types of allergy is commonly attributed to a “western lifestyle”, following the hygiene hypothesis suggested in the 1980s^11^. However, since then it has been clarified that a significant contribution stems from other factors^12,13^. From the environmental standpoint, land use and land cover transformations, on-going climate change, rising CO_2_ levels and temperature can contribute to changes in exposure to pollen and to subsequent changes in allergy prevalence. Therefore, a retrospective assessment of concentrations of allergenic pollen and their trends is important for understanding the epidemiology of pollen allergy and constructing long-term exposure-response models^14,15^.

Apart from the public health-related motivation, information on biological particles in the air can shed light on large-scale changes in biodiversity, ecosystem services, species migration, habitat degradation, planting preferences, etc^16^. Pollen seasonal abundance could serve as one of potential markers of the ecosystem state and composition.

Today, long-term retrospective assessments of pollen concentrations are based on a limited number of long *in-situ* observation time series^17–19^, whose representativeness for other regions is often unclear. The only pollen-related modelling study covering several decades was made for birch and grass without assimilating the observations^20^. It aimed at evaluation of meteorology-driven trends and variability, thus leaving the land use and plant productivity changes out of scope.

Model-based assimilation of observed concentrations of atmospheric tracers can potentially improve both air quality forecasts and retrospective analysis^21–27^. However, practical large-scale and operational applications of data assimilation (DA) technique for air quality are comparatively rare, partly because the classical DA brings limited improvement of model performance, whereas the extended formulations are complicated and costly^21–23,28^. The extended methods are regularly used only for CO_2_ source apportionment, inversion, and attribution^21,29–32^. The CO_2_ emission monitoring, initially mostly based on an inversion of transport matrices^32^, now involves sophisticated variational DA methods, Ensemble Kalman Filter and Smoother, Bayesian inversion, etc^33–35^. An important feature of most CO_2_-related studies is a long temporal scale (window) of assimilation. It reduces the impact of random fluctuations of an otherwise quite homogeneous field of CO_2_ concentration, thus increasing sensitivity of the method to small but systematic sources and sinks.

**Fig. 1:**
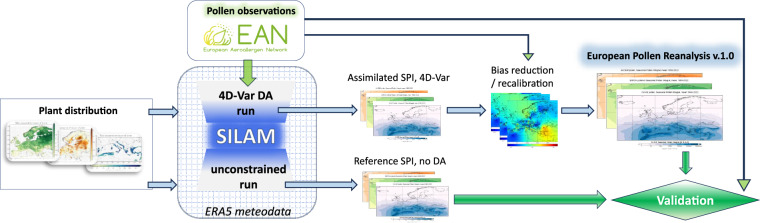
Scheme of the European Pollen Reanalysis.

The goals of the current study are: (i) to perform model-based reanalysis of pollen dispersion for alder (1980–2022), birch (1980–2022), and olive (1985–2022) over Europe, (ii) to evaluate the extended 4-dimensional variational data assimilation (4D-VAR) technique with a long assimilation window for reproducing the pollen season strength, and, finally, (iii) to produce a publicly available long-term Europe-wide pollen reanalysis dataset. The main components and procedures used for generation of the pollen reanalysis are summarized in Fig. 1.

## Methods

### Pollen modelling and data assimilation technology

Modern pollen dispersion models demonstrate quite good skills in representing timing of pollen seasons of trees, as well as variations of pollen concentrations during the season^36–40^. Comparatively simple but efficient parameterizations for pollen production and release were also described long ago^39–42^. The absolute levels of pollen concentration are reproduced much worse. To-date, there is no Europe-wide model for predicting the intensity of the pollen season (e.g., Seasonal Pollen Integral, SPIn^43^). Practically applicable models have been made only for Northern Europe^44^ and Bavaria^45^. Improving the SPIn representation via DA is therefore a high priority task with an important practical outcome.

To-date, there are few practical examples of data assimilation applied to pollen dispersion, whether in operational forecasting or in retrospective analysis. Individual experiments cover a range of approaches, such as pre-processing the model setup using real-time pollen observations, model-measurement fusion and other post-processing, and up to full-scale pollen DA studies^28,38,46^. The first demonstration of 4D-VAR for pollen source inversion was performed with the SILAM model (System for Integrated modeLling of Atmospheric coMposition, http://silam.fmi.fi^47^) within the MACC project (Monitoring Atmospheric Composition and Climate). A more detailed study by Sofiev^28^, hereinafter referred to as S19, expanded the methodology of Vira & Sofiev^27^, hereinafter VS12, and compared efficiency of the extended 4D-VAR and classical DA methods.

### Problem formulation

Addressing arguably the most-uncertain parameter of pollen dispersion models, the reanalysis took the total Seasonal Pollen Integral (SPIn^43^), a time-wise integral of concentration [pollen day m^-3^], as the target parameter for assimilation. For many tree taxa, the total amount of pollen released during a season is related to the previous-season flowering intensity and to conditions during the inflorescence buds formation, which typically occurs during the spring-summer months. The conditions of the current season can only change the timing of the release and, in rare cases, reduce its intensity by destroying the tree inflorescences by a late frost or long intense rain. Under this assumption, a convenient target parameter of the SILAM pollen module is a map of total pollen production $E(i,j,{yr})$: the number of pollen grains released during the whole season per unit area of surface covered by the corresponding tree taxon. Here, *i,j* are grid indices in the corresponding maps, and *yr* is year. This parameter was suggested by the S19 study^28^.

### Input data

The input to the reanalysis includes meteorological data, land use information, plant distribution areas, and pollen observations.

#### Meteorological input

The pollen reanalysis is driven by the meteorological reanalysis ERA5 of the European Centre for Medium-Range Weather Forecasting, ECMWF (http://www.ecmwf.int)^48^. The dataset has been downloaded from the ECMWF archive in a regular lon-lat grid with resolution of 0.25°× 0.25°, which corresponds to the high-resolution products available in the Climate Data Store, CDS (https://cds.climate.copernicus.eu)^48^. The vertical structure of ERA5 consists of 137 hybrid levels, of which SILAM used levels 61–137 (from ~104 hPa down to the surface). The temporal resolution of ERA5 is one hour.

Inconsistencies in wind fields due to reprojection from the reduced-Gaussian grid are handled with the SILAM meteorological preprocessor^49^, which also produced additional quantities characterising the atmospheric boundary layer^50^.

#### Distribution maps of alder, birch, and olive

There is no homogenised dataset quantifying the distributions of alder, birch, and olive. Even the most-comprehensive maps of the European Forest Institute (EFI, https://efi.int/knowledge/maps/treespecies, visited 9 Dec 2023), European Atlas of Forest Tree species (https://forest.jrc.ec.europa.eu/en/european-atlas/, visited 9 Dec 2023)^51^, or the COoRdinate Information on the Environment (CORINE, https://land.copernicus.eu/pan-european/corine-land-cover, visited 13 Mar 2024) do not cover the Russian & Belarussian territory and tend to contain artefacts in areas with poor local data. Birch and alder are not distinguishable in satellite images, where they are just labeled as a mixture of broadleaf deciduous vegetation. Therefore, the distribution of birch was compiled from several sources following the procedure set by Sofiev *et al*.^42^. Areas with good-quality EFI maps were used as-is and extrapolated latitude-wise to poorly described regions using satellite-observed broadleaf forest maps as proxies. The resulting map had a 0.01° × 0.01° resolution. For alder, the tree-specific information is even scarcer than for birch, therefore a generic land use dataset ECOCLIMAP^52^ was used with a fraction of appropriate land use types assigned to alder.

For olive, a both naturally occurring and cultivated tree, with plantations recognizable from high-resolution satellite images, the CORINE database served as the best proxy. This inventory also has a time dimension: the first map of olive plantations was made in 1990 with collection period covering 1980s. After that, the inventory was updated every 6–10 years, thus allowing for a time-resolving source term. However, CORINE covers only a part of Europe and has no data about Africa. Therefore, Africa had to be filled-in from the ECOCLIMAP general-forest maps, with fractions of olive trees roughly determined by visual inspection of satellite images. Last but not least, olive is a popular ornamental plant in Southern Europe (e.g., https://www.gardenista.com/posts/simple-landscaping-ideas-10-genius-gardens-with-an-olive-tree, visited 6 Jun 2024), which was accounted for by assigning a small fraction (<1%) of urban and suburban land use type to be a source of olive pollen. The urban areas were also taken from CORINE, thus accounting for their evolution in time.

**Fig. 2:**
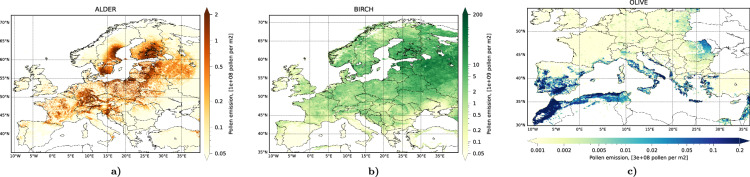
Emission distribution maps of (**a**) alder, (**b**) birch, and (**c**) olive (map from 2020) used in the reanalysis. Unit: see colour bars. Each map shows the multi-annual mean release of pollen grains of the corresponding type per 1 m^2^ of the grid cell area.

For all species, the final step was a multi-annual calibration of the maps as described by Prank *et al*.^53^ aiming at unbiased representation of the SPIn, all over the modelling domain. The result is a distribution of mean annual pollen emission per unit area (Fig. 2).

The difficulties in construction of the distribution maps and the related uncertainties resulted in a forced decision to keep the distribution maps of alder and birch constant throughout the reanalysis, thus relying on DA to reproduce both the short-term and long-term changes in the plant abundance and pollen production.

#### Pollen observations

The standard device for pollen observations in Europe through decades has been the Hirst-type pollen trap^54^, which is one of the longest-used devices in atmospheric composition observations. It is an impactor: the particles are sucked into the inlet orifice at ~10  l min^−1^ flow rate and, due to their inertia, hit a sticky tape located 0.7 mm behind the orifice. The flow rate and the tape position are selected so that >50% of particles of approximately 5 μm of aerodynamic diameter hit the tape and get stuck there. This 5 μm size is considered to be a cut-off of the trap: smaller particles avoid colliding with the tape, whereas practically all particles notably larger than 5 μm get captured. The tape is fixed on a drum rotating with a speed of one revolution per week, which corresponds to about two mm per hour of the tape movement. Upon completion of a weekly cycle, the tape is cut into daily segments and mounted on microscope slides and then particles stuck on the tape are manually counted using a light microscope. The device is simple in construction and affordable, which made it a de-facto standard in aerobiology for over 70 years. The observational procedures and good practices have been homogenised^43,55,56^ and recently brought to the European standard CEN 16868^57^.

A limitation of the Hirst-type devices is the quite tedious manual microscopic analysis of slides collected usually during the previous week, or even earlier. A typical resolution of the produced data is one day, but at a price of additional counting time, 2-hourly data can be generated as well. A higher temporal resolution is not possible without altering the drum rotation speed: the width of the air jet exposing the moving tape with particles approximately corresponds to two hours of the tape motion. The device also has other inherent uncertainties and, sometimes, reliability issues^58,59^.

**Fig. 3:**
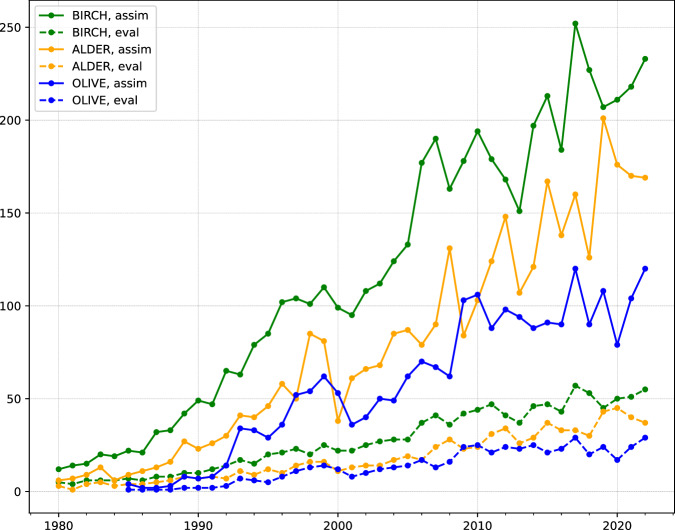
The number of stations used for assimilation and validation for each species over the reanalysis period.

In Europe, most observational groups participate in a voluntary European Aeroallergen Network (EAN), which maintains a common database of observations since 1974 (https://ean.polleninfo.eu/Ean, visited 30 Nov 2023). This reanalysis brought together teams from 34 European countries, thus representing the largest joint effort of the European aerobiological community to date. A full list of participating networks and organisations is provided in the Acknowledgement section. The number of stations available for the reanalysis varied from barely a dozen of sites in the 1970s up to over 300 in 2020 s (Fig. 3), i.e., the number of monitoring sites has increased almost 30-fold compared to the earlier years.

A limitation of the existing observations is the network sparsity in several Eastern European countries and Russia. In addition, the Pollen Information Dienst (PID) network in Germany decided not to participate in the reanalysis. Therefore, in the currently used dataset Germany is represented by the Electronic Pollen Information Network ePIN^60^, which has a comparatively short history of observations, i.e., 5 years for most of the stations, the site in Munich with the time series starting in 1989, and a dense network of 24 stations operated in Bavaria in 2015 within a preparatory project towards ePIN. The ePIN network operates the automatic impactor BAA-500^61,62^, which was among the devices that compared favourably with the Hirst-type impactors within a recent intercomparison campaign^63^. All other sites included in the reanalysis operated Hirst-type devices throughout the considered period.

### SILAM atmospheric composition model

The reanalysis was performed with the SILAM model, v.5.9.1. It is an offline global-to-local chemistry transport model developed for evaluating atmospheric composition and air quality^47^, emergency decision support applications^64^, source inversion problems^26,27^, and analysis of observations^65,66^. SILAM is equipped with a variety of source modules, of which this study used the pollen release module^38,39,67^. Pollen is transported in the air as a chemically inert aerosol, whose mixing and deposition followed the standard SILAM descriptions^50,68,69^.

SILAM AQ predictions have been evaluated in many regional and global studies showing robust performance^49,70–74^. Operational evaluations of its global and regional atmospheric composition and AQ forecasts are available at http://silam.fmi.fi (visited 20 May 2024) for a global comparison against satellite-derived aerosol optical depth, at https://cams2-83.aeroval.met.no/evaluation.php (visited 20 May 2024) for a European evaluation of 6 pollutants against *in-situ* EIONET data, at https://hpfx.collab.science.gc.ca/~svfs000/na-aq-mm-fe/dist/ (visited 20 Sep 2023) for a North-American evaluation of O_3_, NO_2_, PM_2.5_ against *in-situ* sites in Canada and US, and at https://dust.aemet.es/ (visited 20 Sep 2023) for a North-African, Middle East and South-European evaluation of dust against satellite and *in-situ* observations.

The SILAM bioaerosol source module currently includes twelve types of bioaerosols: pollen of alder, birch, grass, olive, five groups of *Artemisia* genera, ragweed, hazel, and a recent extension to nonclassical bioaerosols, such as aphids and ladybirds^38,39,53,67,75,76^.

SILAM is an open-code system (https://github.com/fmidev/silam-model, accessed 8 Dec 2023). The reanalysis was performed with the latest operational release of SILAM v.5.9.1 (DOI 10.5281/zenodo.10351493) available from the above repository.

### Tree pollen source term

All tree pollen sources in SILAM are described by using the same double-threshold heat-sum approach^39,41^ with tree-specific distribution maps and coefficients of the governing equations. It distinguishes between two stages of pollen emission: development of ready-to-fly pollen and its release into the air. The development stage is controlled exclusively by accumulated heat, whereas the release is a function of actual meteorological conditions^39^. The increment of ready-to-fly pollen ${p}_{{rdy}}$ is described as^39^:

1 $$\frac{d{p}_{{rdy}}}{{dt}}=\left[\begin{array}{ll}0: & H < {H}_{{fs}}\ast (1-{\delta }_{H})\\ S{\rm{\phi }}\,{N}_{{tot}}\frac{T-{T}_{{co}}}{\varDelta H}h(T-{T}_{{co}}){p}_{{fs}}\left(\frac{H}{{H}_{{fs}}}\right){p}_{{fe}}(R): & H > {H}_{{fs}},R < 1+{\delta }_{N}\\ 0: & R > 1+{\delta }_{N}\end{array}\right]$$

Here *S(i,j)* is area [m^2^] of grid cell *(i,j)*, *ϕ(i,j)* is fraction of area covered by the corresponding tree crowns, *N*_*tot*_ is amount of pollen released during the full season per unit area fully covered by crowns of the pollen-producing trees [pollen grains] (Fig. 2 shows the product $(\phi \,{N}_{{tot}})$), *T* is temperature [K], either the hourly or the daily mean, depending on the taxon, *T*_*co*_ is cut-off temperature [K], *ΔH* is difference between the heat-sum of the end and the start of the flowering season (*H*_*fe*_, *H*_*fs*_, respectively) [K day] or [K hour], *h*(.) is Heaviside step function (=0 if its argument is negative and =1 otherwise), *p*_*fs*_ and *p*_*fe*_ are probabilities of a single tree to start or end the flowering, *R* is fraction of *N*_*tot*_ released until the current moment, *δ*_*H*_ is uncertainty of heat-sum threshold, and ${p}_{{rdy}}$ is amount of pollen available for release in the grid cell [pollen grains].

Whenever ${p}_{{rdy}}$ > 0, the release rate is^39^:

2 $$E\left(i,j\right)={p}_{{rdy}}\left(i,j\right)\left(1-{e}^{-\frac{\triangle t}{\tau }}\right){f}_{{wind}}\left(U,{w}^{* }\right){f}_{{thr}}\left(q,{q}_{{low}},{q}_{{high}}\right){f}_{{thr}}(P,{P}_{{low}},{P}_{{high}})$$

**Fig. 4:**
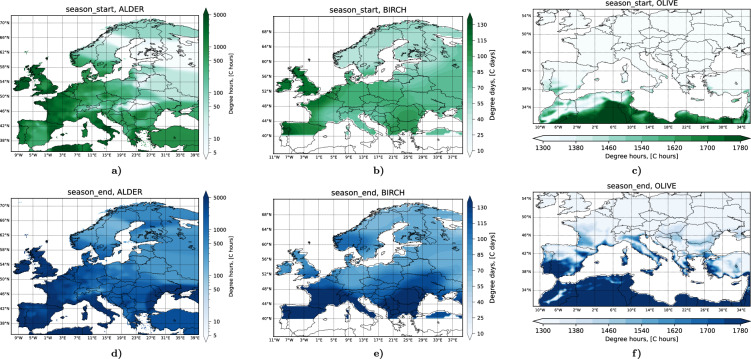
Heat-sum start/end flowering thresholds for alder, birch and olive. No birch trees were assumed south of 40 N.

Here *E* is emission rate in the grid cell [pollen sec^−1^], *τ* is time constant for the exponential decay of the ready-to-fly pollen under normal conditions [sec], *f*_*wind*_ is correction for wind speed *U* [m sec^−1^] and convective velocity scale w* [m sec^−1^], *f*_*thr*_ is threshold correction function applied to relative humidity *q* (thresholds *q*_*low*_ and *q*_*high*_) [%] and precipitation rate *P* (thresholds *P*_*low*_ = 0 and *P*_*high*_) [mm sec^−1^]. Formulas for the components of Equations (1) and (2) are presented in Sofiev *et al*.^39^, coefficients for the specific genera of trees are shown in Table 1, and maps of heat sum thresholds for the start and the end of flowering periods are presented in Fig. 4.

**Table 1:** Parameters of the SILAM pollen source term for trees.

	Alder	Birch	Olive
**Pollen development model**			
Heat-sum *H* type	Hourly mean-T	Daily mean-T	Daily mean-T
Uncertainty of *H*, [%]	10	10	10
Start day of heat accumulation	1 January	1 March	1 January
Cut-off temperature, [°C]	4	3.5	0
Standard pollen release, *N*_*tot*_ [pollen grains m^−2^ yr^−1^]	10^8^	10^8^	3×10^8^
Uncertainty of *N*_*tot*_ [%]	10	10	10
**Pollen release model**			
Shortest release time, *τ*, [hour]	1	1	1
Low-humidity threshold, *q*_*low*_, [%]	50	50	50
High-humidity threshold, *q*_*high*_, [%]	80	90	80
Precipitation_threshold, *P*_*high*_ [mm hour^−1^]	0.5	0.5	0.5
Wind speed saturation level, [m sec^−1^]	5	5	5
Wind speed max scaling	1.5	1.5	1.5
Emission injection height range, [m]	1–50	1–50	2–50
Pollen size, [μm]	22	22	28
Pollen density, [kg m^−3^]	800	800	800

### Setup of the 4D-VAR assimilation procedure

#### Control variable

Similar to S19, this reanalysis uses the extended 4D-VAR technique^27^. We denote the target parameter, or a control variable, as *ξ*, and define the model operator *M* mapping this variable to a unique phase-space trajectory of the model state *x* through *x* = *Mξ*. The mapping is defined over some finite time interval referred to as assimilation window. The vector of observations *y* corresponds to *x* via the observation operator $O$: *y* = $O$*(x)* + *ε*, where *ε* is the observation error. Assuming *ε* to be Gaussian, the maximum likelihood of *ξ* corresponds to a minimum of the cost function:

3 $$J(\xi )=\frac{1}{2}{(y-{Ox})}^{T}{\Phi }^{-1}(y-{Ox})+\frac{1}{2}{(\xi -{\xi }_{b})}^{T}{B}^{-1}\left(\xi -{\xi }_{b}\right)$$

Here *Φ* and *B* are the observational and background covariance matrices, respectively. The first term is a penalty for deviation from observations whereas the second one penalizes the deviation from the initial guess of the control variable.

The parameter deciding the total seasonal pollen release, *N*_*tot*_, has a climatological value specific for each tree type (Table 1). The dependence of *N*_*tot*_ on year and location has been disregarded in the original model^39^, but it is straightforward to introduce it via a correction map. The control variable *ξ*: of Eq. (3) can be set equal to this map and defined as:

4 $$N\left(i,j,{yr}\right)=\xi \left(i,j,{yr}\right){N}_{{tot}}$$

This correction map is unitless and it’s a-priori value is ${\xi }_{b}$ = 1.

There is a physically meaningful restriction, $\xi \ge 0$, which is inconvenient from a practical standpoint because requires conditional minimization procedure. Therefore, in SILAM 4D-VAR, this variable is used in a log-transformed form: $\xi =\exp (\zeta ),\,{\zeta }_{b}=0.$ This transformation is monotonic and does not change the features of Eq. (3), but it eliminates the conditional minimization procedure since the range of meaningful values of *ζ* is $(-\infty ,\infty )$. An evident downside of this transformation is that the optimization can no longer completely switch off the source in a grid cell: the requirement $\xi \ge 0$ is enforced as $\xi  > 0$. which may be too limiting in some cases. In practice, however, false emission source can be made negligibly low, so that its impact does not affect the results.

#### Covariance matrices and measures against over-fitting

We employed the background covariance matrices of VS12 with a few adjustments.

Matrix *B* incorporates standard deviation of the forecast error and spatial correlation between the grid cells. The former is assumed to be constant, while the latter is set through a correlation distance *ρ [m]*:

5 $${corr}({\boldsymbol{r}},{\boldsymbol{r}}{\prime} )=\exp \left(-\frac{{\mathrm{||}{\boldsymbol{r}},r{\prime} \mathrm{||}}^{2}}{{\rho }^{2}}\right)$$

Here, ***r***, ***r***′ are 2-D vectors representing two locations within the 2-D computation grid, ||·|| is the *L*_*2*_ norm of such a vector. Compared to VS12, the following simplifications were made: (i) it is assumed that most of pollen is released and transported within the boundary layer, which is well-mixed, thus eliminating the vertical dimension of the DA problem, (ii) correlation distances latitude- and longitude-wise are assumed to be the same and equal to *ρ*. The first assumption is supported by the near-surface location of pollen sources and the large size of the grains, which keeps them within the boundary layer most of the time. The second one is supported by the VS12 analysis, which showed a barely 10% difference between the meridional and latitudinal correlation distances for sulphur oxides (SOx). For pollen the difference is expected to be even smaller due to the short pollen transport distance and a wide distribution of the sources.

The actual value of the correlation distance *ρ* depends on the transport features and source distribution, but also on temporal averaging. For this reanalysis, *ρ* was determined experimentally, by running the assimilation over several years with different *ρ* values and evaluating the improvement of the model-measurement agreement for non-assimilated stations. The best improvement was found for *ρ* ~ 250 km, but the sensitivity turned out to be quite low. Compared to *ρ* ~ 80 km for SOx emission determined by VS12, the 250 km correlation distance is reasonable: SOx sources are mostly isolated, e.g., power plants, whereas tree pollen production is controlled by the interplay of synoptic-scale meteorological processes, distribution of sources, and major topographic elements^44^.

Specific absolute values of *B* and *Φ* are not relevant: the location of the minimum of the cost function (3) does not depend on the magnitude of the covariances, but rather on their ratio. Therefore, the observational covariance was assumed constant and diagonal, equal to 6 pollen grains m^−3^, close to the Hirst-device detection limit^77^, and the background correlation *B*, (5), was multiplied with 10, thus allowing an order of magnitude variation of *ξ* with minor penalty, i.e., *B* = 1000 ${corr}\left({\boldsymbol{r}},{\boldsymbol{r}}{\prime} \right)$. Both values are at the low end of the corresponding uncertainties, but their ratio is of the correct order of magnitude. Computations showed that with such values, the observation-related term of the cost function is at least 100 times larger than the background, i.e. the minimization is sensitive to even small (1–10%) improvements of the model-measurement distance but disregards the low level noise present in the term (~0.1%).

As a final measure against over-fitting, the iterative minimization procedure of 4D-VAR was monitored with a truncated-iterations watchdog^78^. After every successful adjoint-and-forward cycle, a so-called L-curve^79^ was calculated to check for signs of over-fitting. This method proved efficient in identifying the optimal iteration, after which further minimization iterations were prone to over-fitting. The result of the data assimilation process was set to be equal the value of *ξ* at the optimal iteration step, thus truncating the minimization cycle. The method of truncated iterations was used by Vira *et al*.^26^ and S19, with positive outcome.

#### Assimilation window

The selection of the time-independent control variable suggests that the assimilation window should cover the whole flowering season of each taxon. In Europe, the season gradually propagates from south-west to north-east following the increase of temperature and the melting of snow. Among the considered taxa, alder is the first one to flower, followed by birch and then olives. Time windows that cover their seasons over Europe in all considered years are:

- Alder: 5 January – 31 May
- Birch: 10 March – 1 July
- Olive: 1 April – 31 July

The simulations started on the first day of the heat sum accumulation (Table 1), but the cost function (3) was calculated and minimised only within the assimilation window.

#### Observational data averaging and filtering

The averaging period of the observations for the time-independent control variable could be as long as the assimilation window. However, the mere existence of the pollen season, with the start and the end, makes the problem non-stationary and non-ergodic, i.e., renders averaging inapplicable. Moreover, season-long averaging would require nearly 100% data availability because any value missing from the observations during the season would affect the SPIn and potentially disqualify the time series. However, some averaging was still possible.

The strongest argument in favour of the long averaging period is that it removes high-frequency variations of concentrations, which are not needed for the SPIn assimilation but penalised by RMSE, i.e., the first term of Eq. (3). To demonstrate this, the Eq. (3) is simplified by assuming the observation operator *O* to be an identity operator and the observational covariance matrix $\Phi$ to be a scalar equal to 1. Let’s split both observations and model predictions into their seasonal mean value and high-frequency normalised anomalies: $x=\bar{x}(1+\mu ),{y}=\bar{y}(1+\nu )$, where overbar denotes averaging and $\bar{\mu }=\bar{\nu }=0$, $\bar{{\bar{x}}^{2}}={\bar{x}}^{2}$ and $\bar{{\bar{y}}^{2}}={\bar{y}}^{2}$. Then the RMSE term is transformed to:

6 $$\begin{array}{c}J={(y-Ox)}^{T}{\Phi }^{-1}(y-Ox)=\overline{{(y-x)}^{2}}=\overline{{(\bar{y}(1+\nu )-\bar{x}(1+\mu ))}^{2}}\,\\ \,=\,{\bar{x}}^{2}(1+\bar{{\mu }^{2}})+{\bar{y}}^{2}(1+\bar{{\nu }^{2}})+2\bar{y}\,\bar{x}(1+\overline{\mu \nu })\end{array}$$

Introducing the control variable of assimilation from the Eq.(4): $\,x=\xi z,\,\bar{x}=\xi \bar{z},{z}=\bar{z}(1+\mu )$, one can find the minimum of the error with regard to $\xi$ analytically. Indeed, by substituting *x→z* into the Eq. (6), one obtains a quadratic form with regard to $\xi$, which should be minimised:

7 $$J={\xi }^{2}{\bar{z}}^{2}\left(1+\bar{{\mu }^{2}}\right)+{\bar{y}}^{2}(1+\bar{{\nu }^{2}})-2\xi \bar{z}\,\bar{y}(1+\overline{\mu \nu })\to \begin{array}{c}\min \\ \xi \end{array}$$

Its minimum is:

8 $${\xi }_{{opt}}=\frac{\bar{y}}{\bar{z}}\ast \frac{1+\bar{\mu \nu }}{1+\bar{{\mu }^{2}}}$$

The term $\bar{\mu \nu }$ in the numerator of the ratio (8) is the normalised temporal covariance of observations and model predictions, i.e., the correlation coefficient. Should the time series correlate strongly, their normalised fluctuations would be equal, ${\xi }_{{opt}}$ would be just a ratio of the mean values, and assimilation would lead to an unbiased solution. But if correlation is below unity, the scaling will be smaller, and the assimilation will under-estimate the observed concentrations. The degree of the under-estimation is proportional to the standard deviation of the model output.

**Fig. 5:**
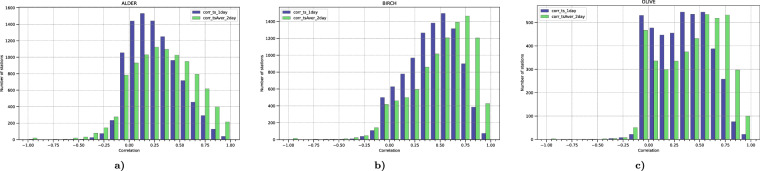
Histogram of correlation coefficients for 1-day (blue) and 2-day (green) mean observations and model predictions. All stations satisfying the completeness requirements (>30 days of data in the given year) are included.

As a compromising solution, an averaging period of two days was selected, and only stations reproduced with a temporal correlation coefficient better than 0.3 were used. Averaging over two days resulted in a very significant increase of temporal correlation (Fig. 5), thus both reducing the bias (8) and increasing the number of stations passing the 0.3 inclusion threshold. Also, at least 30 days of valid observations were required for a particular year for the station time series to be included, and at least 5 of them were required to be non-zero.

The accepted stations were split into assimilation (80%) and evaluation (20%) subsets. The split was semi-random: it was required that spatially isolated stations were always used for assimilation. Therefore, in each year and for each taxon, a list of mandatorily assimilated stations was manually prescribed, after which the remaining stations were split randomly. The distributions were subsequently checked and, if all stations in some regions appeared in the evaluation list, the split was rerun. This requirement helped maintaining the acceptable coverage of the assimilated stations in the pre-1990 period when the network consisted of few stations (Fig. 3).

### Bias reduction: calibration of the mean release strength

To correct the regular low bias of the assimilation output originated from the imperfect model-measurement correlation, after the assimilation, the mean pollen source term strength was calibrated against the whole-period observed SPIn following the procedure of Prank *et al*.^53^.

The SILAM model was run through the whole period with the assimilated season strength and the total multi-annual SPIn was calculated from the co-located observations and model predictions for each station (*SPIn*_*obs*_ and *SPIn*_*mdl*_, respectively). The computations were made only for stations and years with at least 30 daily observations available (no threshold for model-measurement correlation). The relative error *r*_*s*_ was then computed:

9 $${r}_{S}=\frac{{{SPIn}}_{{mdl}}}{{{SPIn}}_{{obs}}}$$

The ratio *r*_*s*_ was subsequently inter-/extrapolated over the whole domain using a Radial-Basis Function with a linear kernel and a smoothing parameter equal to 10. The obtained smooth gridded correction was used as a climatological correction factor to the map of seasonal pollen emission. Computations were performed independently for each tree taxa.

### Setup of the SILAM model runs

Totally, three sets of the SILAM model runs were performed: the first guess run, the data assimilation run, and the final run.

**The first guess (reference) run** was made with the unconstrained model, through the whole period for all species not accounting for any year-to-year variability in pollen production. The run set the reference point for the reanalysis and showed the skill scores to outperform. The setup followed the SILAM operational pollen forecast. The horizontal grid was the same as in the European ensemble of Copernicus Atmosphere Monitoring Service (CAMS): 700 × 420 grid cells, resolution 0.1° × 0.1°, longitude range (25W-45E), and latitude range (30N-72N). The vertical structure consisted of 9 uneven stacked layers, up to 6725 m above the surface: 25 m, 50 m, 100 m, 200 m, 400 m, 750 m, 1200 m, 2000 m, and 2000 m thick. Output included hourly 3D concentrations and 2D dry and wet deposition.

**The DA run** was performed with assimilation of the pollen data as described above generating the set of annual pollen emission correction maps for each year and species. Due to high computational demand of 4D-VAR, the assimilation was performed with a coarser resolution of 0.25° × 0.25° and a vertical with 6 layers with thicknesses of 50 m, 100 m, 400 m, 1000 m, 2000m, and 3000 m. The domain was also reduced to cover the observational network with ~5° margin: horizontal grid of 200 × 168 cells, longitude range (10W-40E), latitude range (30N-72N).

The DA run produced two types of output: the annual emission correction maps for each year and species, and near-surface pollen concentrations. The latter was used to calculate the constant-in-time bias-reducing correction map (9). It did not require additional SILAM computations.

**The final run** used the annual emission intensity correction map from the DA run, extrapolated to the east and linearly downscaled from the DA grid to the source grid, and additionally scaled with the bias-reducing map (9). The rest of the setup was identical to the first guess unconstrained run, thus allowing for a direct comparison.

All simulations used zero lateral boundary conditions and a fully reflective top boundary.

Computations were performed using the SILAM v.5.9.1 (10.5281/zenodo.10351493) with OMP+MPI parallelization (Open Multi-Processing + Message Passing Interface). Assimilation was set year- and species-wise, each using two 128-CPU (Central Processing Unit) nodes of a supercomputer. The runtime for both the DA and the bias elimination run were between 1 and 3 hours depending on the speed of the 4D-VAR convergence and the simulation grids.

## Data Records

The European Pollen Reanalysis v.1.1 is freely available for the public. The metadata and the link to the main archive^80^ have been registered as 10.57707/fmi-b2share.85841086f9db46b882d750eaa9e42515 and PID: http://hdl.handle.net/11304/4766ae3f-f7cb-4967-b601-9b0479600e98 A direct link to the data is: https://european-pollen-reanalysis.lake.fmi.fi/index.html.

The **input** data that were used for constructing the reanalysis have been described in the Methods section.

The **output** data records of the Pollen Reanalysis v.1.1 are the following (for more details, see the above metadata DOI). The **output horizontal grid** is the same as that of the European ensemble of Copernicus Atmosphere Monitoring Service: 700 × 420 grid cells, Mercator projection, resolution 0.1° × 0.1°, longitude range (25W-45E), and latitude range (30N-72N). The **output vertical** consists of 9 stacked layers, up to 6725 m above the surface and being 25 m, 50 m, 100 m, 200 m, 400 m, 750 m, 1200 m, 2000m, and 2000m thick. Supplementary 2D fields are provided in the **data assimilation horizontal grid**, Mercator projection, resolution 0.25° × 0.25° and 200 × 168 grid cells, longitude range (10W-40E), latitude range (30N-72N).

All fields cover the full reanalysis period of 1980–2022.

**Fig. 6:**
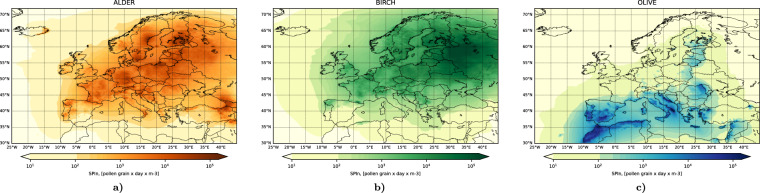
Mean multi-annual SPIn, [pollen day m^−3^]. The Seasonal Pollen Integral for each pollen type is computed as a sum of daily-mean near-surface concentrations, separately for each year. The obtained annual integrals are averaged over the whole period 1980–2022.
- 3D pollen hourly concentrations in the output horizontal grid and output vertical
- 2D hourly dry and wet deposition fields in the output horizontal grid
- 2D annual pollen productivity correction fields, in the DA grid
- 2D seasonal footprint area from the assimilated stations in the DA grid

The following variables are provided in the dataset:

- 2D near-surface pollen hourly concentrations in the output horizontal grid (example in Fig. 6).

All files are in the netCDF4 format, closely following the CF-1.3 convention (https://cfconventions.org, visited 3 Dec 2023), tested for viewing with GrADS v.2.0, Python 3.7 netCDF4 library, and NASA PanoPly netCDF/HDF/GRIB data viewer (https://www.giss.nasa.gov/tools/panoply visited 3 Dec 2023).

## Technical Validation

### Validation of the DA procedure

**Fig. 7:**
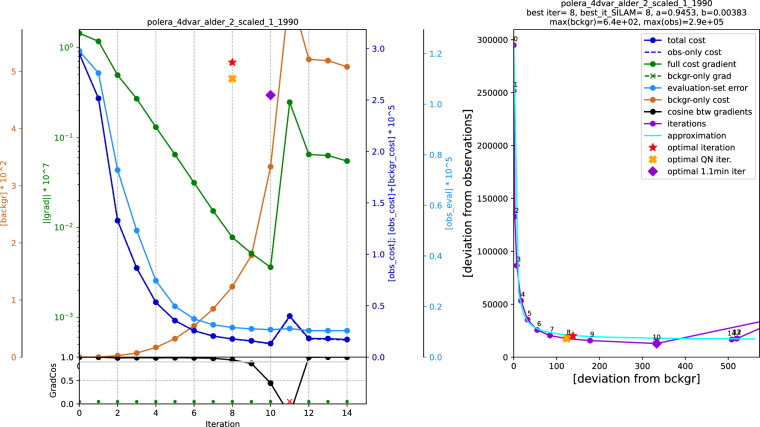
Diagnostics of the DA iterations: error reduction and L-curve-based stopping criterion for alder in 1990. Left-hand panel: changes in the squared deviation from observations (first term in Eq. (1), dark blue line), squared deviation from the background (second term in Eq. (1), brown line), norm of the gradient of the full cost function (1) (green line), squared deviation from the evaluation subset of the observations (light blue line), and cosine of an angle between two sequential iteration steps (black line at the lower panel), all as functions of the 4D-Var iterations (iteration 0 corresponds to the background state). The lower panel also shows the iterations used for fitting the analytical approximation of the L-curve (green dots at the bottom) – and those excluded from the fitting due to suspected instability of the optimiser. Right-hand panel: L-curve, a dependence of the squared model deviation from the assimilated observations on the deviation of the control variable from the background (the first and the second terms of the Eq. (1), respectively): magenta broken line is plotted iteration-wise, light blue curve is its smooth analytical approximation with a hyperbolic function. In both panels, the red star shows the stopping criterion determined from the analytical L-curve approximation as 5% threshold of the initial slope; the nearest iteration is then considered as the optimal one. The orange cross denotes the first estimate made during the calculations with a simplified approximation procedure, whereas the violet diamond shows the first iteration when the full cost function reaches a 1.1 level of its overall minimum during the optimization.

Online validation during the assimilation iterations is based on the truncated-iteration and L-curve technology^78,79^. It verifies the cost-function reduction and checks for potential over-fitting. A typical example is shown in Fig. 7 for alder assimilation for the year 1990. This diagnostic is primarily used for stopping the iterations not relying on absolute tolerances or other formal criteria of the minimization routines, but on more physically grounded considerations of regularization via truncated iterations^78,79^. These are based on the assumption that the large eigenvalues of the gradient matrix will be driving the initial quick reduction of the cost function along the corresponding phase dimension(s), whereas the smaller eigenvalues will start driving the process only after the fast decay is over (iteration 6 in Fig. 7). At that point or soon after it the iterations can be stopped. The actual criterion for stopping the iterations in the current reanalysis was the 20-fold reduction of the initial slope of the L-curve.

The strength of the over-fitting and representativeness of the assimilation and evaluation stations are visible from the comparison of dark and light blue curves from the left-hand panel of Fig. 7. In the provided example, 20× reduction of deviation from the assimilated stations corresponds to 10× reduction of error in the evaluation stations, with a marginal sign of error growth at the iteration 10. This confirms a good correspondence of the assimilation and evaluation stations and manifests negligible over-fitting in this case. A large distortion of the procedure after the iteration 9 reflects the complexity of the problem and a limited stability of the quasi-Newton optimizer: upon completion of the initial well-defined descend, the optimiser becomes sensitive to small errors and noise in the data. The ultimate task of the truncation procedure is to stop the iterations before it happens.

**Fig. 8:**
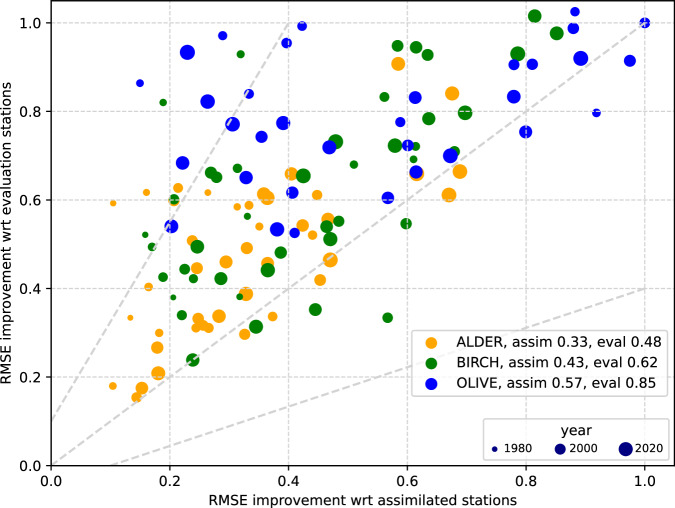
Summary of L-curve diagnostics for the whole reanalysis. X-axis: RMSE for assimilated stations of the optimal 4D-VAR iteration normalised with that of the 0^th^ iteration (unconstrained run), y-axis: RMSE for evaluation stations at the optimal iteration normalised with that of the 0^th^ iteration. On both axes, 1.0 means no improvement due to the data assimilation. Points above the 1:1 line show the years where improvement was higher for assimilated stations than for evaluation ones: large displacement shows an overfit. Two side dashed lines show the slope of 0.5 and 2 with an offset of −0.1 and 0.1, respectively. The size of the dots is the smallest for 1980 and gradually grows towards 2022. Legend also shows mean RMSE improvement (a fraction of RMSE left after DA).

The summary for all years and tree species of the L-curve diagnostics is provided in Fig. 8. As one can see, the most challenging task was assimilation of the olive pollen data: the improvement of performance was limited or sometimes absent. On the upside, the over-fitting was insignificant: RMSE was not much worse for evaluation stations than for assimilated stations, i.e. the assimilation procedure remained consistent even when the improvement was limited. There are several reasons for that behaviour. Firstly, comparatively few stations observing olive pollen are distributed over a very long but narrow area along the Mediterranean coast. Atmospheric transport between these sites is limited, which complicates the selection of evaluation stations: every station is unique. Secondly, the inter-annual variation of olive pollen production is low due to human management of the agricultural crop: irrigation, fertilization, intensification, etc., i.e. the signal to assimilate is low^81^. Nevertheless, for about two thirds of the years, the improvement reached or exceeded ~20%, which is a good outcome for such a complicated case.

Assimilation of alder and birch was more efficient: the RMSE improvement was more than a factor of two for alder (down to 48% of initial value) and over a third for birch for the evaluation stations. The over-fitting was very limited: it exceeded a factor of 2 ± 0.1 for only two early years for birch (small green dots outside the range marked by grey lines) when the network density was not sufficient to constrain the model.

### Inter-annual SPIn variation

**Fig. 9:**
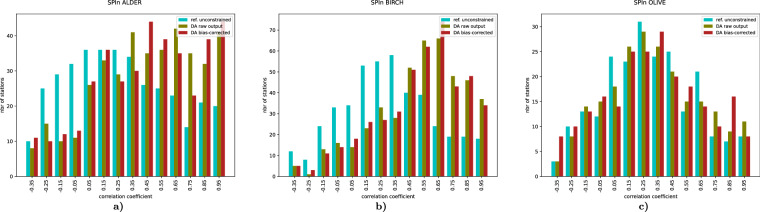
Histograms of correlation coefficient between the observed and predicted/assimilated inter-annual SPIn variations. Three cases are considered: unconstrained reference run, raw output of the DA run, and bias-corrected final run. All years and all stations with >3 years of valid observations are included.

The primary target of the assimilation process was to resolve the inter-annual variation of the SPIn, Eq. (4). The effect of the DA can be described in terms of correlation coefficient of the observed and predicted SPIn (Fig. 9). Comparison of the panels of Fig. 9 reveals three signals: (i) even an unconstrained run (cyan-coloured bars) shows certain skill in reproducing the inter-annual SPIn variation, (ii) assimilated time series demonstrate much higher correlation with the observed SPIn variability, (iii) some stations remain poorly reproduced in all runs. The first observation reflects the effect of the transport conditions during the season on the SPIn, which appeared to be significant, in line with the earlier hindcast computations^20^. The second one confirms efficiency of the selected DA procedure. The third one shows the areas of improvement: the most-probable reason for persistently poor representation of some stations is a deficiency of regional/local sources, which lead to representativeness issues for the corresponding stations.

**Fig. 10:**
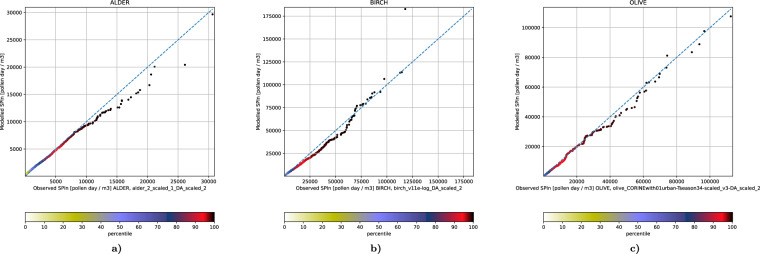
Quantile plots for assimilated SPIn. The plot is obtained by an independent sorting of the observations and the corresponding model predictions, thus disregarding their temporal co-location and only accounting for the relation between the distribution functions of the observed and predicted concentrations.

The assimilated SPIn fields also exhibit excellent frequency distributions. The plots in Fig. 10 show practically perfect quantiles for all species.

### Efficiency of the post-DA bias reduction

As shown in the Methods section, assimilation of the imperfectly correlating data leads to a negative bias in the results. This bias has been corrected by the post-DA recalibration of the emission maps following the procedure of Prank *et al*.^53^ (Fig. 1). Its efficiency was verified by computing the ratio of the observed and predicted SPIns over the whole period of 43 years at each station:

10 $${r}_{{st}}=\frac{\sum {x}_{{st}}}{\sum {y}_{{st}}}$$

The summation in Eq. (10) is performed over all valid observed daily data of the station, and the corresponding model predictions. Full co-location, in both space and time, ensured consistency and comparability of the multi-annual SPIns.

**Fig. 11:**
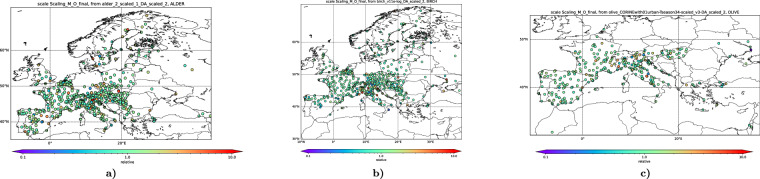
Ratio of observed and modelled multi-annual SPIn.

Figure 11 shows that the model predictions for all species are in-average unbiased: except for a few outliers, the vast majority of the stations report a ratio of modelled and observed SPIn close to unity. This is in agreement with the quantile plots of Fig. 10.

### Temporal correlation of daily concentrations

**Fig. 12:**
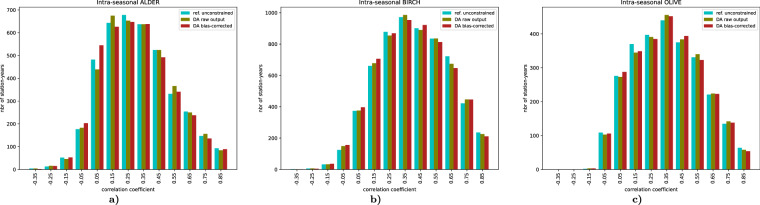
Histograms of temporal correlation coefficient between observed and predicted 2-daily-mean pollen concentrations, 1980–2022. The coefficient is computed for each station and for each year.

The assimilation of the target variable of the reanalysis, SPIn, has a limited and non-equivocal effect on the day-to-day variations of concentrations within the season. Indeed, changing the regional pollen emission intensity alters the long-range transport patterns, thus affecting both source and receptor locations. As SILAM has been calibrated to an inter-annually constant release rate, this reanalysis opens new possibilities for model improvement. However, in the current study the impact of improved SPIn to intra-seasonal model performance was negligible (Fig. 12). The final fields, with the assimilated SPIn and the corrected bias, show similar skills as the initial run, thus confirming that the short-term features of the season remain essentially unconstrained.

### Areas constrained by the observations

**Fig. 13:**
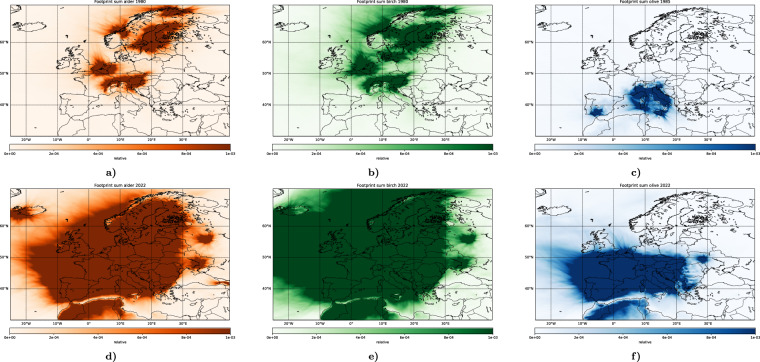
Areas observed by the active stations in 1980 (upper row, 1985 for olives) and 2022 (lower row), [relative]. The presented variable is the sum of footprints of all active stations throughout the growth and flowering season 1 January–31 July.

Due to varying number of stations (Fig. 3), area constrained by the data is different each year. Secondly, the PID network did not participate in the reanalysis, potentially reducing the reliability of the results in northern Germany. However, even the comparatively sparse network was efficiently used by the assimilation procedure constraining the emission not only in the vicinity of the stations, but also in the regions covered by footprints of the available stations (Fig. 13 upper row). For instance, even in 1980 when no German networks were operational, pollen release in north-western part of the country was constrained by the data from Belgian stations, whereas its southern part was visible for Austrian sites. Nevertheless, one has to be careful in interpretation of the obtained results during this decade because the well-observed regions in Europe were isolated from each other, and the current assimilation procedure provided only limited information about their pollen levels. With a gradual growth of the network, the coverage improved, so that after ~1990 most of Europe was covered by the station footprints and constrained in the reanalysis (Fig. 13 lower row).

**Fig. 14:**
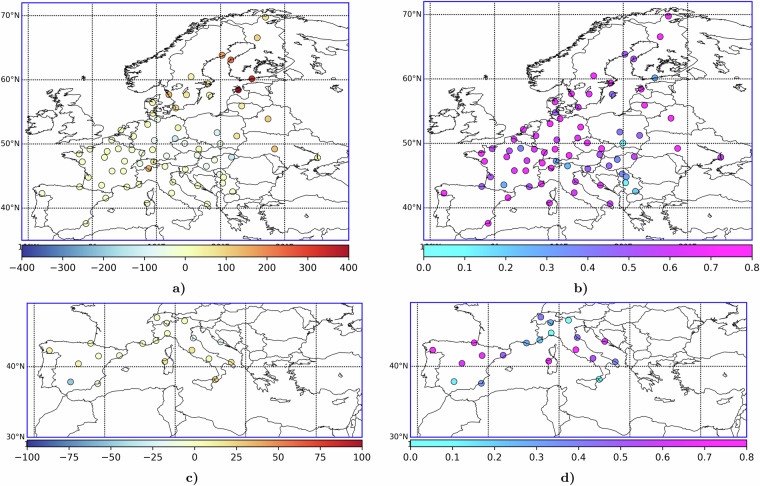
Bias (left column, panels **a,c**) and correlation coefficient (right column, panels **b,d**) of the SILAM birch (upper row, panels **a,b**) and olive (lower row, panels **c,** **d**) forecasts in 2022, as evaluated by Copernicus Atmospheric Monitoring Service^86^. Adopted from CAMS283-2023 report, available for unrestricted use from the CAMS Web portal^86^.

In view of the limited network coverage in some periods and regions, existing model evaluation exercises can be used to estimate uncertainty of the unconstrained model simulations. In particular, birch model was evaluated in a series of studies^37,39,42,67,82–85^ showing robust performance. An important addition to these studies is a recent model evaluation report of Copernicus Atmospheric Monitoring Service (CAMS)^86^, which evaluated 11 European models, all equipped with the SILAM pollen emission module, against the EAN pollen monitoring data provided to CAMS for model evaluation purposes. The report confirms the small bias and very high correlation of the SILAM unconstrained forecasts for birch and olive in 2022 (Fig. 14).

## Usage Notes

The reanalysis output includes, apart from the near-surface pollen concentrations, the 3-D fields of hourly pollen concentrations, thus allowing for nested high-resolution computations over any part of Europe. Therefore, high-resolution model-based assessments can use the re-analysis as a boundary condition.

Long-term re-analysis is a convenient source for various trend estimates. However, a common problem (frequently under-estimated) is that the trend of availability of the observations can significantly affect the analysis. For pollen reanalysis, the number of monitoring stations varies greatly depending on the specific year (Fig. 3). Therefore, SILAM computations over several regions in 1980s and, to a less extent, 1990s are practically unconstrained. To facilitate the interpretation of the reanalysis and its application to long-term studies, it is accompanied with annual footprints of the available monitoring stations (Fig. 13). As shown by Sofiev *et al*.^87^, footprints of monitoring stations provide valuable information on the network fidelity. Illustrating the phenomenon, the maps of Fig. 13 show the areas constrained by the data assimilation in 1980 and 2022. A full set of years is provided as a part of the reanalysis dataset. Areas not covered by the footprints cannot be constrained by the current DA technology with the available monitoring network during the particular year. Consequently, the pollen release over these areas is equal to its regional long-term mean. Unconstrained areas should be considered with care and excluded, for instance, from a season severity trend analysis.

The reanalysis followed the protocol suggested by the VS12 and S19 studies, which also pointed out the possibility of operational forecasting with assimilation of real-time pollen measurements. To date, the scarcity of such observations in Europe is limiting its implementation at the continental scale, but regional applications can be already feasible.

While making use of the reanalysis, one should keep in mind that the main attention was paid to the overall severity of the pollen season, i.e., the SPIn. The reanalysis did not aim to resolve day-to-day concentrations, which remained the essentially unconstrained SILAM model predictions, with their strengths and weaknesses (Fig. 12). Therefore, usage of the reanalysis for tasks sensitive to daily or hourly values (e.g., clinical trials) can be recommended only in combination with the actual pollen monitoring data. Details of the monitoring data requesting procedure can be obtained from https://www.ean-net.org/, (visited 7 Dec 2023).

## Data Availability

The reanalysis has been performed with the latest operational release of the SILAM model v.5.9.1, which is freely available from https://github.com/fmidev/silam-model. The version actually used for the reanalysis is released by Kouznetsov & Tyuryakov^88^.
